# Supplementary material for: Identification of photoperiod-induced specific miRNAs in the adrenal glands of Sunite sheep (Ovis aries)
Source: Front Vet Sci. 2022 Jul 22;9:888207. doi: 10.3389/fvets.2022.888207 (PMC9354845; doi:10.3389/fvets.2022.888207)
Supplement: Supplementary file 1 [file Data_Sheet_1.ZIP › Supplementary file/miRDeep2 file.zip!/miRDeep2 file/Know_Structure/LP42A2/oar-mir-30a.pdf]

miRBase precursor : oar-mir-30a
Total read count : 126397
oar-miR-30a-5p read count : 119813
oar-miR-30a-3p read count : 6584
remaining reads : 0

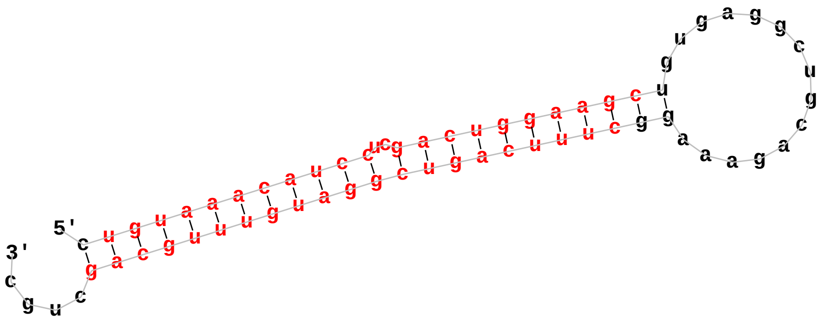

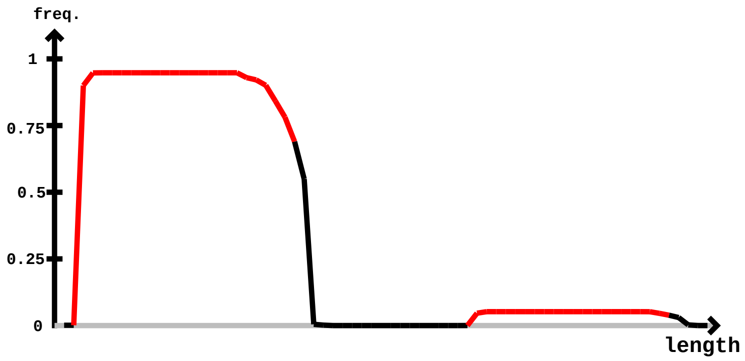

oar-miR-30a-3p

oar-miR-30a-5p

5'- cuguaaacauccucgacuggaagcugugaggcugcagaaaggcuuucagucggauguuugcagcugc -3' exp

((((((((((((..(((((((((((...............)))))))))))))))))))))))....	reads	mm	sample
cuguaaacauccuUgacu...	5	1	seq
cuguaaacauccucgacu...	1	0	seq
cuguaaacauccuUgacug..	1	1	seq
cuguaaacauccucgacugg...	5	0	seq
cuguaaacauccuUgacugg...	2	1	seq
cuguaaacauccucgacugga..	28	0	seq
cuguaaacauccuUgacugga..	1	1	seq
cuguaaacauccuUgacuggaa...	2	1	seq
cuguaaacauccucgacuggaa...	12	0	seq
cuguaaacauccuUgacuggaag..	2	1	seq
cuguaaacauccucgacuggaag..	14	0	seq
cuguaaacauccuUgacuggaagc...	4	1	seq
cuguaaacauccucgacuggaagc...	25	0	seq
cuguaaacauccuUgacuggaagcu..	25	1	seq
cuguaaacauccucgacuggaagcu..	21	0	seq
cuguaaacauccucgacuggaagcug...	1	0	seq
.uguaaacauccuUgacu...	1993	1	seq
.uguaaacauccucgacu...	316	0	seq
.uguaaacauccuUgacug..	787	1	seq
.uguaaacauccucgacug..	273	0	seq
.uguaaacauccucgacugg...	1251	0	seq
.uguaaacauccuUgacugg...	1308	1	seq
.uguaaacauccuUgacugga..	1323	1	seq
.uguaaacauccucgacugga..	6060	0	seq
.uguaaacauccuUgacuggaa...	1289	1	seq
.uguaaacauccucgacuggaa...	6370	0	seq
.uguaaacauccucgacuggaag..	7521	0	seq
.uguaaacauccuUgacuggaag..	3966	1	seq
.uguaaacauccucgacuggaagc...	14833	0	seq
.uguaaacauccuUgacuggaagc...	2783	1	seq
.uguaaacauccuUgacuggaagcu..	37030	1	seq
.uguaaacauccucgacuggaagcu..	26358	0	seq
.uguaaacauccucgacuggaagcug...	28	0	seq
.uguaaacauccuUgacuggaagcug...	74	1	seq

```
                                         oar-miR-30a-3p
 oar-miR-30a-5p
cuguaaacauccucgacuggaagcugugaggcugcagaaaggcuuucagucggauguuugcagcugc

.uguaaacauccuUgacuggaagcugu........................................    34      1     seq
.uguaaacauccucgacuggaagcugu........................................    9       0     seq
..guaaacauccuUgacugg...............................................    5       1     seq
..guaaacauccucgacugga..............................................    8       0     seq
..guaaacauccuUgacugga..............................................    19      1     seq
..guaaacauccucgacuggaa.............................................    8       0     seq
..guaaacauccuUgacuggaa.............................................    10      1     seq
..guaaacauccuUgacuggaag............................................    42      1     seq
..guaaacauccucgacuggaag............................................    13      0     seq
..guaaacauccucgacuggaagc...........................................    30      0     seq
..guaaacauccuUgacuggaagc...........................................    52      1     seq
..guaaacauccucgacuggaagcu..........................................    262     0     seq
..guaaacauccuUgacuggaagcu..........................................    5106    1     seq
..guaaacauccuUgacuggaagcug.........................................    263     1     seq
..guaaacauccucgacuggaagcug.........................................    3       0     seq
..guaaacauccuUgacuggaagcugu........................................    168     1     seq
...uaaacauccuUgacuggaagcu..........................................    24      1     seq
...uaaacauccucgacuggaagcu..........................................    18      0     seq
...uaaacauccuUgacuggaagcug.........................................    14      1     seq
...uaaacauccuUgacuggaagcugu........................................    9       1     seq
....aaacauccucgacuggaag............................................    1       0     seq
....aaacauccuUgacuggaagcug.........................................    2       1     seq
....aaacauccuUgacuggaagcugu........................................    1       1     seq
.........................................gcuuucagucggauguuuA.......    1       1     seq
.........................................gcuuucagucggauguuuAc......    4       1     seq
.........................................gcuuucagucggauguuuAca.....    8       1     seq
.........................................gcuuucagucggauguuuAcag....    6       1     seq
.........................................gcuuucagucggauguuuAcagc...    5       1     seq
..........................................cuuucagucggauguuu........    4       0     seq
..........................................cuuucagucggauguuug.......    4       0     seq
..........................................cuuucagucggauguuuA.......    16      1     seq
..........................................cuuucagucggauguuuAc......    568     1     seq
..........................................cuuucagucggauguuugc......    140     0     seq
..........................................cuuucagucggauguuugca.....    110     0     seq
..........................................cuuucagucggauguuuAca.....    758     1     seq
..........................................cuuucagucggauguuugcag....    281     0     seq
..........................................cuuucagucggauguuuAcag....    766     1     seq
..........................................cuuucagucggauguuugcagc...    1063    0     seq
..........................................cuuucagucggauguuuAcagc...    2057    1     seq
..........................................cuuucagucggauguuugcagcu..    75      0     seq
..........................................cuuucagucggauguuugcagcug.    1       0     seq
...........................................uuucagucggauguuug.......    1       0     seq
...........................................uuucagucggauguuuA.......    1       1     seq
...........................................uuucagucggauguuugc......    11      0     seq
...........................................uuucagucggauguuuAc......    43      1     seq
...........................................uuucagucggauguuugca.....    3       0     seq
...........................................uuucagucggauguuuAca.....    12      1     seq
...........................................uuucagucggauguuuAcag....    24      1     seq
...........................................uuucagucggauguuugcag....    23      0     seq
...........................................uuucagucggauguuuAcagc...    144     1     seq
...........................................uuucagucggauguuugcagc...    192     0     seq
...........................................uuucagucggauguuugcagcu..    249     0     seq
...........................................uuucagucggauguuugcagcug.    9       0     seq
............................................uucagucggauguuuAcagc...    3       1     seq
............................................uucagucggauguuugcagc...    2       0     seq
```
